# Relationship between chemical shift value and accessible surface area for all amino acid atoms

**DOI:** 10.1186/1472-6807-9-20

**Published:** 2009-04-02

**Authors:** Wim F Vranken, Wolfgang Rieping

**Affiliations:** 1 Protein Databank Europe, European Bioinformatics Institute, Cambridge, UK; 2 Department of Biochemistry, University of Cambridge, Cambridge, UK

## Abstract

**Background:**

Chemical shifts obtained from NMR experiments are an important tool in determining secondary, even tertiary, protein structure. The main repository for chemical shift data is the BioMagResBank, which provides NMR-STAR files with this type of information. However, it is not trivial to link this information to available coordinate data from the PDB for non-backbone atoms due to atom and chain naming differences, as well as sequence numbering changes.

**Results:**

We here describe the analysis of a consistent set of chemical shift and coordinate data, in which we focus on the relationship between the per-atom solvent accessible surface area (ASA) in the reported coordinates and their reported chemical shift value. The data is available online on .

**Conclusion:**

Atoms with zero per-atom ASA have a significantly larger chemical shift dispersion and often have a different chemical shift distribution compared to those that are solvent accessible. With higher per-atom ASA, the chemical shift values also tend towards random coil values. The per-atom ASA, although not the determinant of the chemical shift, thus provides a way to directly correlate chemical shift information to the atomic coordinates.

## Background

Nuclear Magnetic Resonance (NMR) spectroscopy provides structural information on an atomic level and is, together with X-ray crystallography, the leading technique for structure elucidation: about 15% of all the protein and nucleic acid structures deposited at the wwPDB [1,2] were solved by NMR. The most prevalent NMR information used to calculate these structures are inter-atomic distances determined by the Nuclear Overhauser Effect (NOE). However, it has long been known that the chemical shift value of an atom is highly sensitive to its local chemical environment [3], and that it could be a highly informative NMR parameter when determining or validating structures. This effect has been exploited using the chemical shifts of backbone atoms to determine protein secondary structure elements [4-6] and dihedral angles [7-9]. More recently, databases that contain chemical shifts from the BioMagResBank (BMRB) [10] were used in conjuction with their corresponding atomic coordinates from the wwPDB to determine tertiary protein structures from chemical shifts [11-13], and to determine protein flexibility [14,15]. Methods to determine chemical shift values from a sequence or coordinates [16-18] or based on empirical algorithms [19,20] exist longer. As these methods are knowledge-based, they often depend on the content and quality of the archives used in creating their knowledge database. Chemical shifts, however, are values that are calculated relative to a reference frequency. This reference frequency is not always correctly set by the experimentalist; in this case, the chemical shift values are consistently offset. Some computational approaches have attempted to 're-reference' the original chemical shift values to obtain more accurate measures, a step that can be crucial to get good results. On the other side of the computational spectrum, ab initio methods that determine the chemical shift from atomic coordinates by quantum mechanical calculations hold great promise to provide accurate values, especially for heavy atoms [21-24]. However, these methods are still computationally too demanding to use in practical day-to-day structure calculations.

The solvent accessible surface area (ASA), which is calculated from the atomic coordinates, is often generated on a per-residue basis. However, other studies suggest that per-atom ASA values provide a more meaningful and precise measure for use in analysis and structure prediction, especially for residues with longer sidechains [25]. In this study, we combine per-atom ASA values with their chemical shift values, for all atoms in all amino acids. The analysis is based on 1959 BioMagResBank entries which were carefully linked to corresponding coordinate data from the wwPDB. We show that the per-atom ASA, as calculated from structure coordinates by the program ASC [26], adds an informative new dimension to the chemical shift data. Atoms with zero per-atom ASA have a significantly larger chemical shift dispersion and often have a different chemical shift distribution compared to those that are solvent accessible. With higher per-atom ASA, the chemical shift values also tend towards random coil values. The per-atom ASA, although not being the determinant of the chemical shift, does provide a way to directly correlate chemical shifts to a property calculated from the atomic coordinates.

## Results and discussion

### Result graphs

All generated plots showing the relation between the chemical shift data and the per-atom ASA are available online from: 

The link *list of the included BMRB entries *on this page connects to a list of all included BMRB entries. For each BMRB entry included in the analysis, a detailed page is available with entry-specific information about the BMRB entry and its link to a corresponding wwPDB code.

The link *original *connects to the data described in this article. Note that the per-atom ASA can only be calculated for heavy atoms, and protons are assigned the same ASA level as the heavy atom they are covalently bonded to. Two types of plot are available:

1. The *exposure *data describes the direct correlation between the chemical shift value of an atom and its associated per-atom ASA value as calculated from the coordinates. These graphs are also available in colour-coded versions where the colour of individual data points designates a particular parameter (*e.g*. the atom is part of a paramagnetic protein, *etc*.).

2. The *correlated *data describes the correlation between the chemical shift value of a heavy atom (*e.g*. CA) and its covalently connected protons (*e.g*. HA). The points in these graphs are colour-coded by the ASA value of the heavy atom or by the secondary structure of the residue they are part of.

In all cases, subdivided graphs are available where only atoms are shown that are part of a residue with a particular secondary structure.

The available data is too extensive to describe in detail, so specific examples are discussed to highlight the usefulness of these graphs. It is clear that the per-atom ASA, as calculated from the coordinates, will not always accurately reflect the real solvent accessibility of the atom in solution. In the following discussion, however, we assume that on average this relationship holds true. This also means that it is likely that some outliers and the spread of values in the graphs is at least partially caused by the uncertainty in the relationship between the per-atom ASA and the real solvent exposure.

### Selected examples

#### Shift to exposure

**Figure 1:**
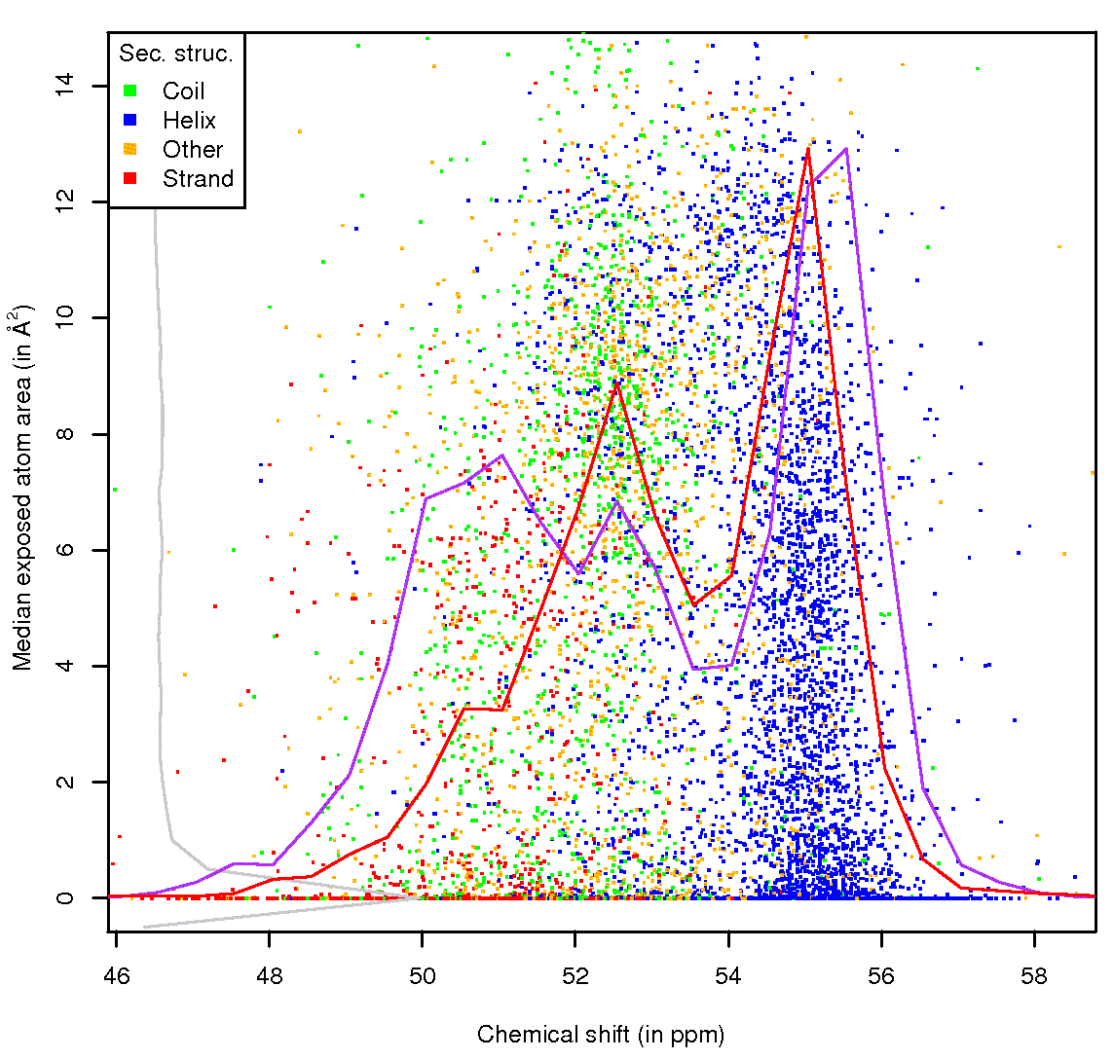
**Ala CA exposure, by secondary structure**. Chemical shift versus ASA values for the CA atom of alanine, with data points coloured by the secondary structure of the residue. Scaled frequency polygons are shown for chemical shifts (for atoms that are exposed (red line) and buried (purple line, zero per-atom ASA)) and for the exposure values (grey).

**Figure 2:**
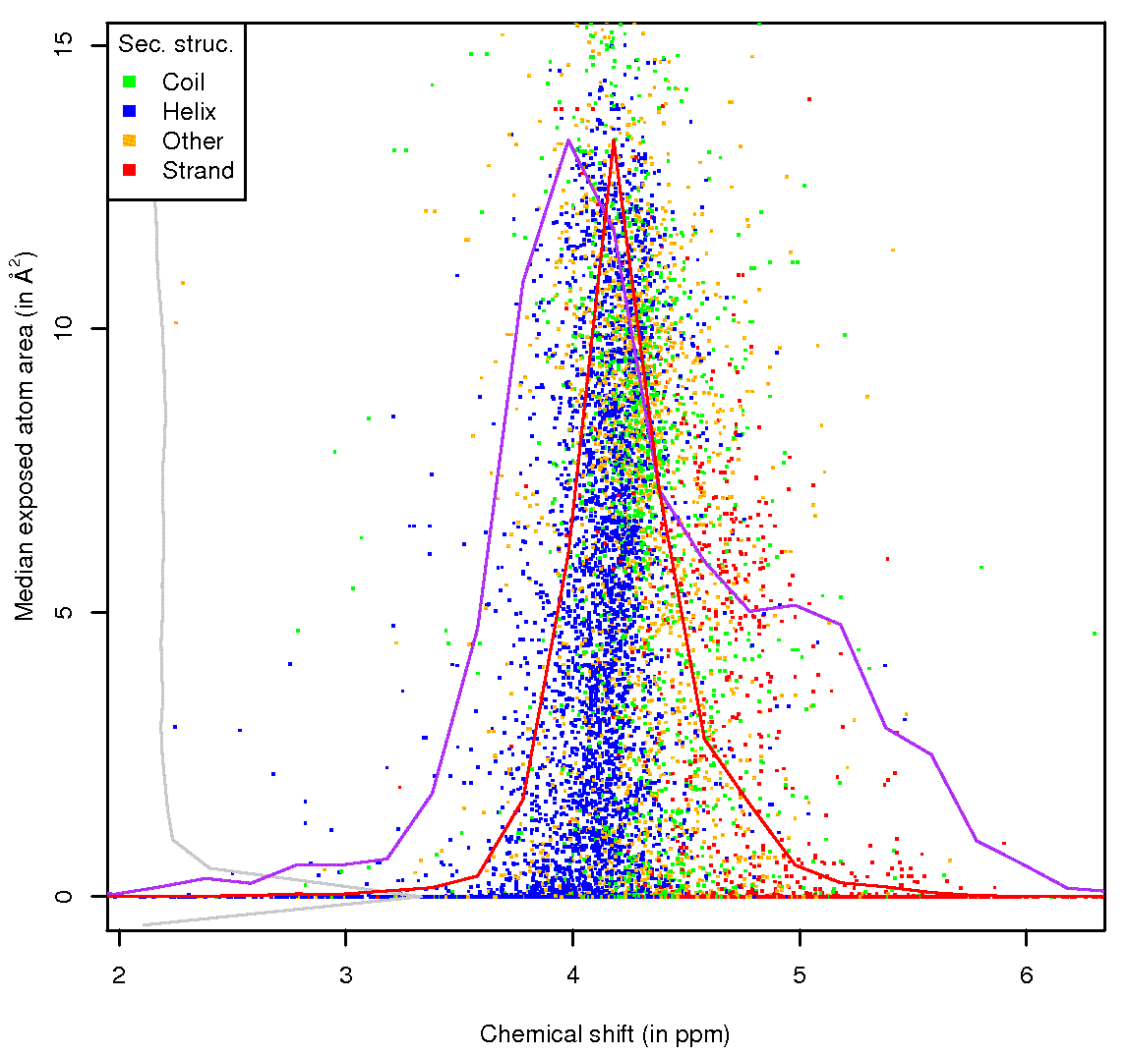
**Ala HA exposure, by secondary structure**. Chemical shift versus ASA values for the HA atom of alanine, with data points coloured by the secondary structure of the residue. Scaled frequency polygons are shown for chemical shifts (for atoms that are exposed (red line) and buried (purple line, zero per-atom ASA)) and for the exposure values (grey).

The first example shows the separation of secondary structure elements for the HA and CA atoms (Figures 1, 2) of alanine, and confirms that the information in the graphs reflects the well known effects of secondary structure [4], as well as the previously reported effect of solvent exposure [27] on the chemical shift. In the CA plot, the secondary structure influence is obvious, with the chemical shift values of CA atoms in helical secondary structure around 55 ppm. The *β*-sheet values are at lower ppm (around 50.5 ppm), with the values for random coil and other secondary structure spread in between. Also noticeable is the decrease in the spread of the chemical shifts with increased per-atom ASA. There is especially a clear change from very low ASA values (below 1 Å^2^) to higher ones. Also, the distributions of the chemical shifts for atoms that are exposed (red line) and completely buried (purple line) are very different. This shows that it is possible to relate the per-atom ASA directly to different chemical shift characteristics for an atom. The reason for this difference is probably that CA atoms in secondary structure elements are more likely to be buried in calculated coordinates, although similar differences are present within secondary structure element distributions (data available online). Finally, the distribution of the exposure points (grey) shows that the density is the highest at zero per-atom ASA.

Similar trends are present in the same type of plot for the HA atom. In this case the helical values are at lower ppm while the *β*-sheet values occur at higher ppm. Interestingly, the average chemical shift value for HA atoms in a helix increases with higher ASA value (the helix data points slant towards the right at higher ASA), while the opposite effect occurs for *β*-sheet HA atoms. The values thus end up close to the random coil chemical shifts (4.34 ppm in case of the alanine HA [28]) at the highest ASA values. This trend confirms the effect of solvent accessibility [27] on HA, CA and CB chemical shifts in secondary structure elements. Also note the overall trend of decreasing spread of the chemical shift values with increasing per-atom ASA, and the very different distribution of chemical shift values for atoms that are buried and exposed.

It is also apparent from these graphs that some atoms have excessive per-atom ASA values. This is because in some cases, especially when the structure was solved by X-ray crystallography, residue coordinates are missing in the PDB file. These points were retained because they reflect the original data and occur only rarely.

**Figure 3:**
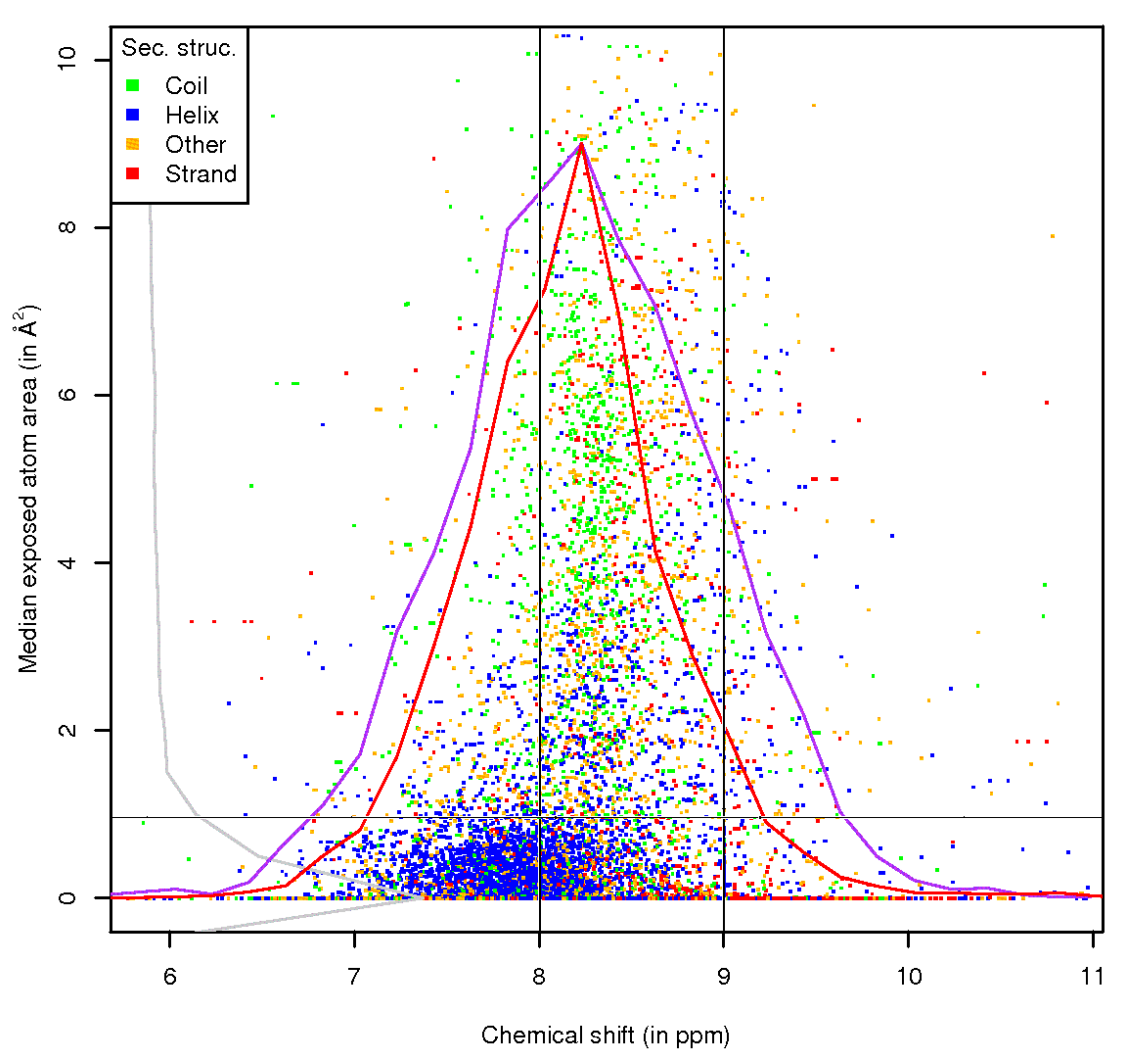
**Ala H exposure, by secondary structure**. Chemical shift versus ASA values for the H atom of alanine, with data points coloured by the secondary structure of the residue. Scaled frequency polygons are shown for chemical shifts (for atoms that are exposed (red line) and buried (purple line, zero per-atom ASA)) and for the exposure values (grey).

For the amide proton of alanine (Figure 3), the chemical shift distribution for buried atoms is again much wider than for exposed atoms. There are also differences within the exposed atom range: above an per-atom ASA value of around 1 Å^2 ^the chemical shift values are mostly between 8 and 9 ppm, with the mean chemical shift value distinctly higher than 8 ppm. Below 1 Å^2^, however, the mean value is clustered around 8 ppm (this is also evident from the shape of the frequency polygon for the exposed atoms). This illustrates that increased solvent exposure of the amide proton results in complex changes in hydrogen bonding and atom shielding, which reflect on the chemical shift value.

**Figure 4:**
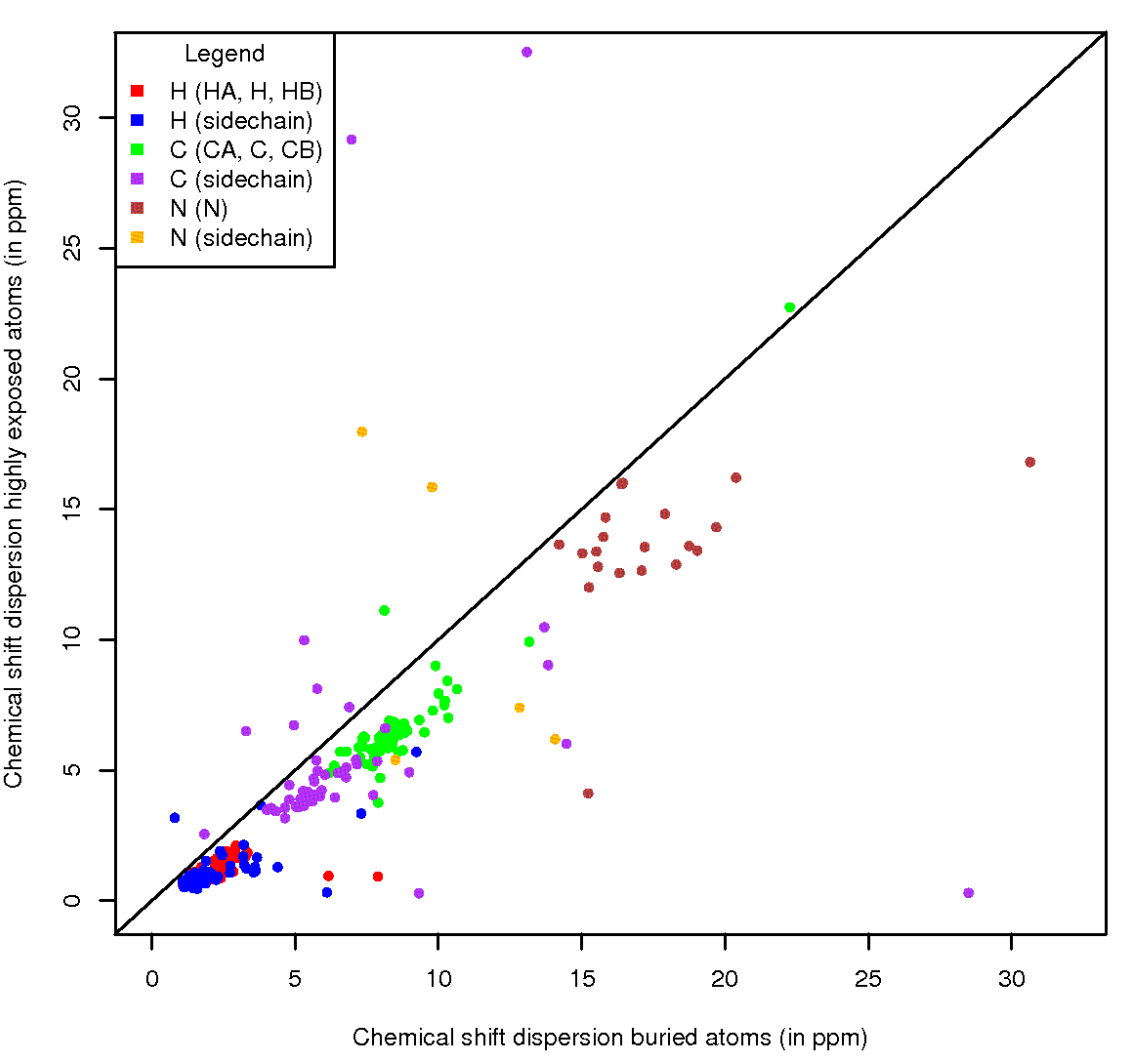
**Shift dispersion by exposure**. The average chemical shift dispersion for highly exposed atoms (based on per-atom ASA) versus the chemical shift dispersion for buried atoms. Data points are coloured by atom type.

These figures show the trend that atoms with high per-atom ASA (highly exposed) have a smaller chemical shift dispersion than atoms with very low per-atom ASA (buried). To quantify this as a general characteristic, we plotted, for each atom in each residue, the average chemical shift dispersion for highly exposed atoms to the dispersion for buried atoms (Figure 4). In general, and especially for backbone atoms, the trend is confirmed. For protons in particular the chemical shift dispersion for buried atoms tends to be double that of highly exposed atoms. Only for very few atoms is this relationship reversed, mostly for atoms that are almost always highly exposed (*e.g*. Arg CZ, NH1, NH2). The arginine carbonyl is the prime exception, with an unusual set of exposed points at higher ppm (data available online).

#### Parameter dependence

**Figure 5:**
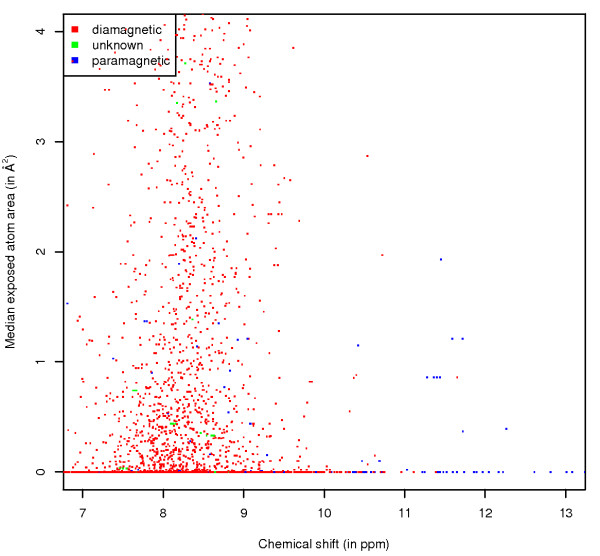
**His H exposure**. Chemical shift versus ASA values for the H atom of histidine, with data points coloured by the paramagnetic status of the protein.

**Figure 6:**
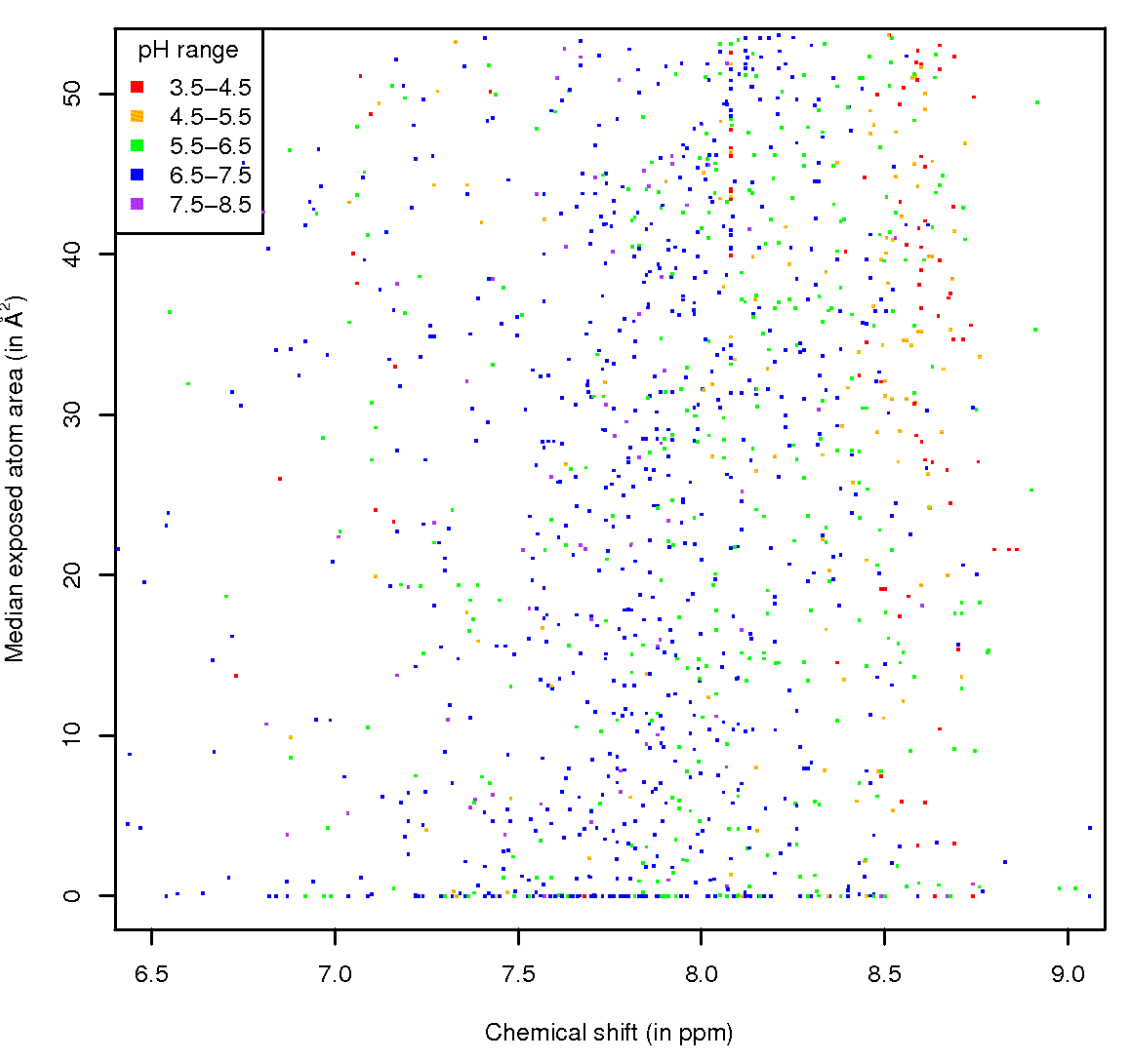
**His HE1 exposure**. Chemical shift versus ASA values for the HE1 atom of histidine, with data points coloured by the pH range at which the protein was studied.

All these graphs were also generated with the data points coloured by different sets of parameters (the corresponding link on the web pages is given between brackets): paramagnetic status of the protein (*Paramagnetic*), the chemical shift referencing molecule that was used (*Referencing*), the pH range (*pH*), whether an entry's origin is from a structural genomics project (*StrucGenomics*) and by anonymised lab origin (*Laboratory*). These subdivisions show some well-known trends, for example most of the unusual chemical shift values for the amide protons for histidine (Figure 5) are observed in paramagnetic proteins (this is also the case for the HB protons (see website)). As a final example, the effect of pH can be seen on the HE1 atom of histidine, with the values recorded at lower pH shifted, on average, to higher ppm (Figure 6).

#### Correlated shift to exposure

**Figure 7:**
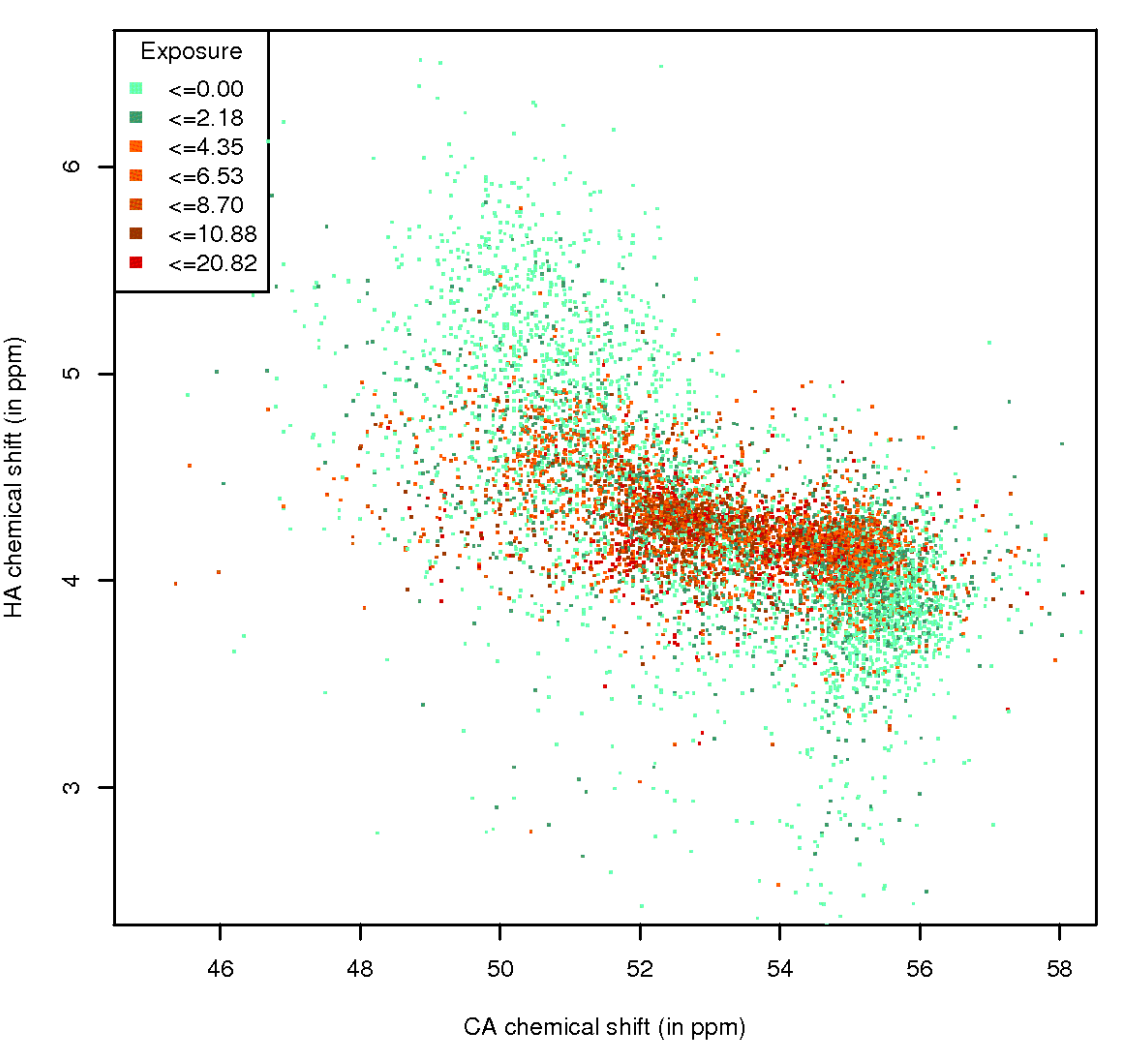
**Ala CA-HA shift correlation, by ASA**. Correlation between the chemical shift values of the CA and HA atoms of alanine, with data points coloured by the ASA of the CA atom.

**Figure 8:**
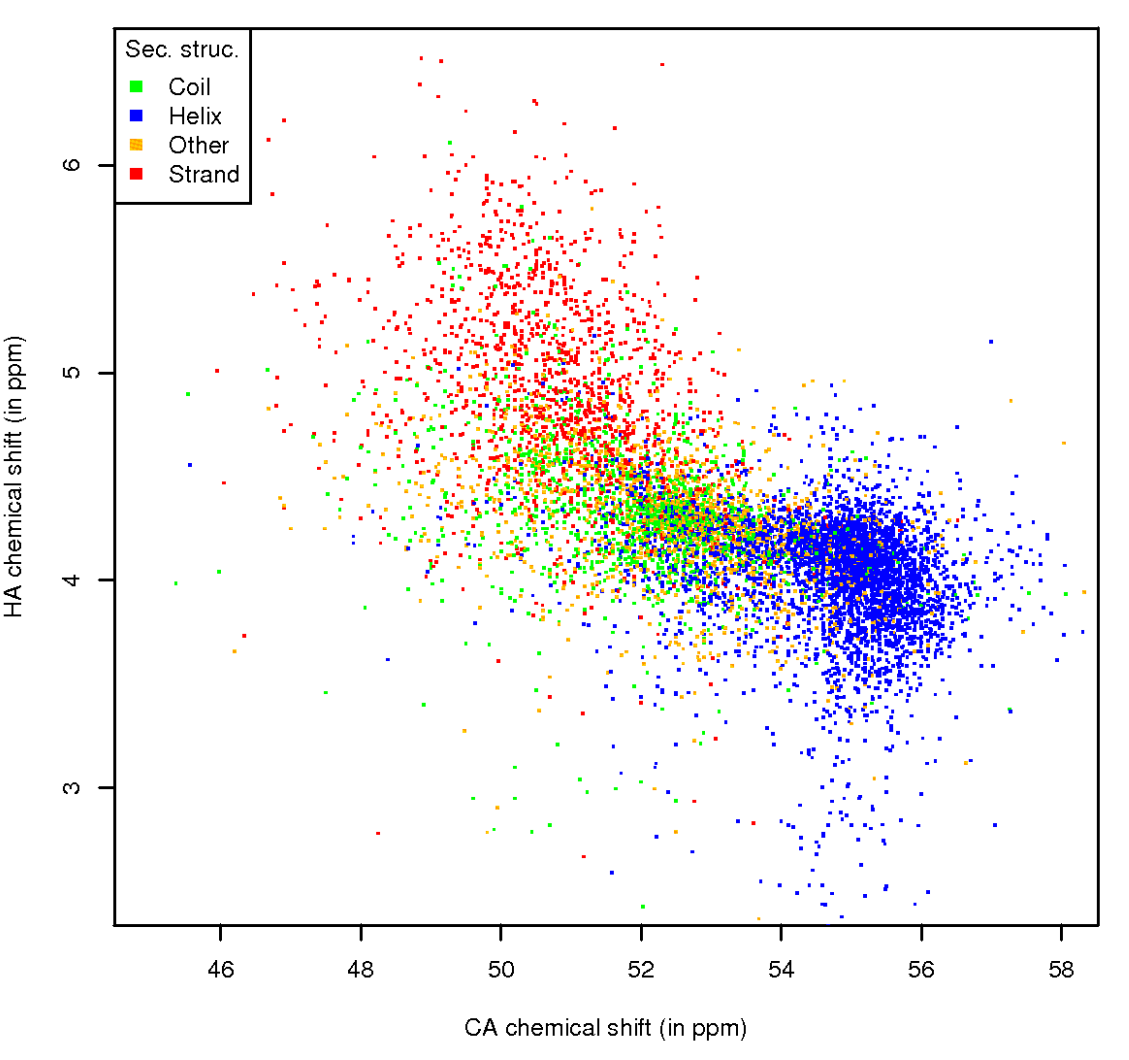
**Ala CA-HA shift correlation, by secondary structure**. Correlation between the chemical shift values of the CA and HA atoms of alanine, with data points coloured by secondary structure.

The trends described here are even clearer when the per-atom ASA values are displayed on plots that show the correlation between chemical shift values of the heavy atom and their covalently bonded protons, if both chemical shifts are available. This information is shown for the CA and HA atoms for alanine (Figure 7). The area of combined CA/HA shifts with a high per-atom ASA is limited to the central region, and the areas where the CA atom is buried are clearly identifiable. These buried regions directly correspond to secondary structure areas (Figure 8).

## Conclusion

Correlating the chemical shift values with the per-atom ASA as determined from the coordinate data adds a new dimension to the chemical shift data. The described plots are very informative and show well-known trends (like the secondary structure dependence of the CA chemical shift). More importantly, two conclusions can be drawn from this data:

1. With increasing per-atom ASA, as determined from the coordinates, the chemical shifts of especially the protons tend towards their random coil value.

2. For most atoms the chemical shift distribution for buried atoms is significantly different from exposed atoms, and there are chemical shift value ranges that indicate the corresponding atom is buried (*i.e*. has zero or very low ASA). This is especially relevant when the chemical shifts of the heavy atom can be combined with their proton shifts.

Since the per-atom ASA is directly calculated from the coordinates, and since it relates differently to the chemical shift depending on its value, it is a parameter that can be used to directly relate coordinates to chemical shifts. For example, two generic methods could be developed based on the above conclusions. First, the chemical shift range for atoms with high per-atom ASA is relatively small and their chemical shift values tends towards random coil. The exposed atoms for a particular protein should therefore, on average, tend towards random coil values. For proteins with reported chemical shifts and coordinates, this effect should make it possible to determine whether the chemical shifts are offset by a certain amount (because of incorrect chemical shift referencing). Secondly, it should be possible to extract chemical shift ranges that indicate with a high probability whether an atom is buried. This information could then be used to validate structures (*i.e*. are atoms that should be buried exposed?), or even employed in structure calculations as an additional constraint, which should be of great assistance in helping globular structures converge in calculations based on chemical shifts only.

Prior to the per-atom ASA values used in this study we did employ per-residue ASA. Although it was recently reported that with increasing per-residue ASA the spread of the chemical shift values for the HA, CA and CB atoms decreases [27], we found that the per-residue ASA did not produce the same quality of results as using the per-atom ASA, especially for amino acids with a longer side-chain like lysine.

Interestingly, the per-atom ASA can be predicted with good accuracy from sequence alone [25], which can make the conclusions of this analysis also useful in applications where the coordinates are not available (for example when validating the chemical shift referencing for proteins with unknown structure).

Because of missing or uncertain coordinate data, the uncertainty that exists between the calculated per-atom ASA and the real atom exposure in solvent, and chemical shift referencing problems [29-31], the spread of the data points is still wider than what would be possible with more accurate data. In another study (Rieping, personal communication), we have attempted to use a generic method based on per-atom ASA to re-reference chemical shift values, which does result in less outliers in the graphs shown in this study. More accurate coordinate data, for example by recalculation with the latest protocols [32], should also improve the calculated per-atom ASA. However, the only reliable way to improve analyses such as this is by ensuring that the available data archive is more accurate. This again stresses the importance of depositing accurate coordinate and chemical shift data, as well as the relevant metadata with regard to the conditions in which the NMR data was recorded. We therefore strongly support the drive to collect more and better experimental data together with the coordinates [33].

## Methods

### Data selection

Archived chemical shift data and reference information from the BMRB [10] and molecule and remediated coordinate data from the wwPDB [1,2] was read into the CCPN data model [34,35] and made consistent with each other in a process similar to the one described previously for the analysis of distance constraint data [36]. For each BMRB entry, a list of related PDB entries was extracted from the BMRB archive, and metadata about the entry was extracted (*e.g*. lab of origin). From this list, the most accurate PDB entry was chosen: if structures solved by X-ray crystallography were available, the entry with the highest resolution was chosen, otherwise the most recent entry determined by NMR was picked. In each case, consistency between the sequence information in the BMRB entry file and the PDB file was ensured, and in case of homologous sequences, chemical shift data related to residues substituted in the BMRB sequence in relation to the PDB one was ignored. If major problems were encountered during the linkage process, either in matching up the BMRB and PDB sequences or with handling the chemical shift data, the entry was ignored.

### Data analysis

The per-atom ASA was calculated from the coordinate data using the ASC software. This software calculates ASA values for the heavy atoms based on their coordinates. For protons the ASA value of the directly bonded heavy atom was used. No provisions were made for missing coordinates or residues, which can lead to excessive ASA values in some cases, as noted before [25]. Secondary structure assignments were calculated using STRIDE [37]. If problems were encountered executing ASC or STRIDE on a PDB entry, or if the resulting information did not directly match up to the data stored in the CCPN framework, the entry was ignored.

Custom-written Python [38] scripts stored the values from the ASC and Stride analysis in PDB-entry specific Python dictionaries so they could be easily retrieved once calculated. In case of NMR ensembles, the median value over all models was taken as the representative for the per-atom ASA. For the per-residue secondary structure, the element that occurred most often for a residue in all the models was selected. This process was executed on 2403 BMRB entries and resulted in 1959 valid CCPN projects where the BMRB information was connected to a unique PDB entry. In total 1632 unique PDB entries were used, that is, although some BMRB entries are linked to the same PDB entry, the chemical shift data was not always recorded in the same conditions and is, therefore, worth including. The inclusion of overlapping data also does not affect the plots and conclusions that can be drawn from them (results not shown). Detailed information on each BMRB entry is available from: 

In many cases, not all original shifts were used in the analysis. This can be due to minor problems with matching the chemical shift information to atoms (*e.g*. tryptophan residues are, based on the coordinate data, often created without HE1 atoms, in which case these shifts cannot be linked).

### Graph preparation

The graphs showing the chemical shift and per-atom ASA values were created with the R software [39] from custom-written Python scripts using the RPy [40] module. In case of multi-colour plots, the order in which the points are plotted was randomised to better represent the data. HTML pages to combine the information were created by custom-written Python scripts.

The frequency polygons in the plots relating the per-atom ASA to chemical shift value were scaled make an optimal comparison of shape possible. The data available online lists the relative scaling factors.

To create the plot showing the exposure based chemical shift dispersion, we first defined (for each atom in each residue) the per-atom ASA value below which 99% of all data points fall. This region was divided into 10 equally spaced 'bins'. The chemical shift dispersion of the points encompassed in each bin was defined as the chemical shift range between the 2.5% percentile to the 97.5% percentile, and thus encompasses 95% of all points within that bin. The average of this range for the 5 bins with highest exposure then defines the chemical shift dispersion for highly exposed atoms, the lowest bin the dispersion for buried atoms.

## Authors' contributions

WV carried out the data preparation, analysis and graph generation. WR participated in interpretation of the results and manuscript drafting. All authors read and approved the final manuscript.
